# Multipool-CEST and CEST-based pH assessment as predictive tools for glioma grading, IDH mutation, 1p/19q codeletion, and MGMT promoter methylation in gliomas

**DOI:** 10.3389/fonc.2024.1507335

**Published:** 2024-12-20

**Authors:** Xinli Zhang, Jue Lu, Xiaoming Liu, Peng Sun, Qian Qin, Zhengdong Xiang, Lan Cheng, Xiaoxiao Zhang, Xiaotong Guo, Jing Wang

**Affiliations:** ^1^ Department of Radiology, Wuhan Union Hospital, Tongji Medical College, Huazhong University of Science and Technology, Wuhan, China; ^2^ Department of Clinical & Technical Solutions, Philips Healthcare, Beijing, China

**Keywords:** glioma, IDH, 1p/19q codeletion, MGMT, pH assessment

## Abstract

**Objectives:**

To comprehensively and noninvasively predict glioma grade, IDH mutation status, 1p/19q codeletion status, and MGMT promoter methylation status using chemical exchange saturation transfer (CEST)-based tumor pH assessment and metabolic profiling.

**Methods:**

We analyzed 128 patients with pathologically confirmed adult diffuse glioma. CEST-derived metrics based on tumor regions were obtained using five-pool Lorentzian analysis and pH_weighted analysis. Histogram features of these metrics were computed to characterize tumor heterogeneity. These features were subsequently employed for glioma grading and molecular genotyping of IDH, 1p/19q and MGMT. Logistic regression analysis was used to predict the grade and IDH genotypes. The diagnostic performance was evaluated using receiver operating characteristic (ROC) curves and area under the curve (AUC) analysis.

**Results:**

The DS, MT and pH_weighted differed significantly between grade II and III, as well as grade III and IV. The amide, NOE, pH_weighted and MTR_3.5_ showed significantly differences within IDH genotypes. Regression models achieved the highest AUC for differentiating grade II from III (0.80, 95% CI: 0.64-0.91), grade III from IV (0.83, 95% CI: 0.74-0.90), and IDH mutant from wild status (0.84, 95% CI: 0.77-0.90). MT and pH_weighted metrics were the only indicators for identifying 1p/19q codeletion in grade II and grade III gliomas, respectively. MT 90th percentile (0.87, 95% CI: 0.65-0.98) and pH_weighted 25th percentile (0.83, 95% CI: 0.56-0.97) showed the best performance, respectively. The MTR_3.5_ was the only indicator which can distinguish MGMT promoter methylation and unmethylation gliomas, within MTR_3.5_ 90th percentile performed best (AUC = 0.79, 95% CI: 0.61- 0.91).

**Conclusion:**

CEST-based tumor pH assessment and metabolic profiling demonstrated promising potential for predicting glioma grade, IDH mutation status, 1p/19q codeletion, and MGMT genotype.

## Introduction

Gliomas are the most common primary brain tumors, characterized by high mortality and morbidity rates (1). According to the 2021 World Health Organization (WHO) Central Nervous System (CNS) classification, adult-type diffuse gliomas are categorized into astrocytomas (isocitrate dehydrogenase mutant [IDH-mt], 1p/19q non- codeletion), oligodendrogliomas (IDH-mt, 1p/19q codeletion), and glioblastomas (IDH wild-type, [IDH-wt]) (2). IDH-wt gliomas are classified as grade IV, oligodendrogliomas as grade II to III, and astrocytomas range from grade II to IV. Glioma grading influences treatment approaches, with high-grade gliomas typically managed by maximal surgical resection followed by adjuvant radiotherapy and chemotherapy, while low-grade gliomas are treated based on the extent of resection and patient factors such as age to determine postoperative adjuvant therapy (3). The new classification guidelines highlight the importance of genotypes and molecular characteristics. Research indicates that patients with 1p/19q codeletion respond better to radiotherapy and chemotherapy, resulting in improved prognosis (4). Additionally, O-6-methylguanine-DNA methyltransferase (MGMT) promoter methylation predicts a better response to temozolomide and enhances survival (5). However, molecular typing often relies on pathological diagnosis, which is invasive, prone to sampling errors, and costly.

MRI is the most commonly used preoperative diagnostic tool for gliomas. Grade IV gliomas frequently exhibit ring enhancement on T1-weighted images, whereas grade II and III gliomas typically show no enhancement, making it challenging to distinguish these grades on imaging. Most studies focus on the comparison between low-grade gliomas (grade II) and high-grade gliomas (grades III and IV), or between lower-grade gliomas (grades II and III) and higher-grade gliomas (grade IV), while the identification of grade III gliomas remains relatively vague. Diffusion-weighted imaging (DWI) has been used to predict MGMT promoter methylation and 1p/19q codeletion (5, 6). However, apparent diffusion coefficient (ADC) measurements are often based on subjective regional delineation, and the heterogeneity of gliomas may introduce selection bias in region of interest settings. Some studies have found that the methylated MGMT promoter type exhibited larger ADC values, while others reported no differences between methylated and unmethylated types (7, 8). Dynamic susceptibility contrast (DSC) and dynamic contrast-enhanced (DCE) imaging have also demonstrated value in predicting MGMT promoter methylation and 1p/19q codeletion, but both methods require contrast agent injection (9–12).

The degree of tumor metabolism is often positively correlated with malignancy. High-grade gliomas exhibit vigorous cell proliferation, angiogenesis or vascular disruption, and an accumulation of more acidic metabolic byproducts in the extracellular space. Persistent hypoxia, increased glycolysis, and heightened acidity in tumors can affect tumor invasiveness and alter gene expression (13, 14). Therefore, characterizing tumor metabolism and the acidity of the tumor microenvironment is a feasible method for grading and predicting molecular subtypes.

Chemical exchange saturation transfer (CEST) imaging is an MRI technique that enhances the detection of low- concentration biomolecules by exploiting the chemical exchange properties between molecules and water protons (15). The exchange rates of certain protons are pH-dependent, making this technique useful for assessing tissue pH, which is crucial for evaluating the tumor microenvironment (16). Previous studies have demonstrated that CEST imaging of amine protons in glutamine molecules can be used as a noninvasive pH-weighted MRI technique for human and preclinical investigations of malignant gliomas (17). Amide proton transfer (APT) imaging is a relatively mature CEST technology, with amide protons in tissues serving as the primary source of the APT signal (18). Studies have shown that APT imaging holds potential for the differential diagnosis, grading, molecular typing, and prognostic evaluation of gliomas (19–24). However, APT imaging based on magnetization transfer asymmetry analysis can overlook confounding factors, including intrinsic semi-solid magnetization transfer (MT) asymmetry and low-field relayed nuclear Overhauser effect (NOE) signals. Methods such as multi-pool Lorentzian analysis and inverse Z-spectrum analysis have been proposed to enhance CEST quantitative analysis (25, 26). Multi-pool Lorentzian analysis decomposes the Z-spectrum into five components: amide, NOE, amine, DS, and MT. Amide and amine represent mobile proteins/peptides and creatine, respectively. The DS signal is related to water proton concentration and tissue relaxation time, while the MT signal originates from immobile macromolecules. The NOE signal comes from the aliphatic and olefinic components of various metabolites, including mobile proteins, peptides, and lipids. Multi-pool Lorentzian analysis has demonstrated potential value in glioma grading and the diagnosis of IDH and 1p/19q genotypes (25, 27).

In this study, we quantitatively describe glioma metabolism and the pH characteristics of the tumor region based on CEST imaging using multi-pool Lorentzian and pH analyses. We explore their value in assessing glioma grade, IDH mutation, 1p/19q codeletion, and MGMT promoter methylation. Additionally, histogram analysis was employed to better characterize tumor heterogeneity.

## Materials and methods

### Patient cohort

**Figure 1:**
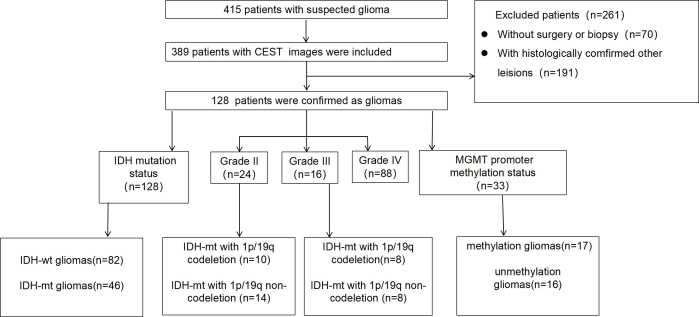
The participant enrollment flowchart.

This study received approval from the Institutional Review Board. Between January 2023 and March 2024, 415 consecutive patients suspected of having gliomas who underwent preoperative CEST MRI examinations were enrolled. The inclusion criteria were: (1) histologically diagnosed adult-type diffuse gliomas, and (2) age >18 years. **Figure 1** illustrates the participant flowchart.

### Data acquisition

Scans were performed using a 3TIngenia CX Philips scanner with an 80 mT/mgradient, 200 mT/m/s slew rate, and a 32-channel head coil. Routine structural MRI included T1-weighted images before and after Gd enhancement, and T2-FLAIR images, with a total acquisition time of 10 minutes. A custom-developed CEST sequence based on 2D multi-offset, single-slice, single-shot turbo spin echo (TSE) was applied to the maximum cross-sectional areas of the tumors with the following acquisition parameters: radiofrequency (RF) saturation power, 0.9 µT; saturation duration, 3,000 ms; TR = 5000 ms, TE = 14 ms, field of view = 200 × 200 mm^2^, voxel of 2.5 × 2.5 × 4 mm^3^, compressed sensing acceleration factor of 4, and flip angle 90 degrees. RF saturation was performed with 2 parallel RF transmission channels (through a body coil) driven by the RF amplifiers in a time‐interleaved fashion. By combining 2 amplifiers, each operating at 50% duty cycle, RF saturation at 100% duty cycle was achieved. The 64 offsets in order were 0, ± 0.25, ± 0.5, ± 0.75, ± 1, ± 1.25, ± 1.5, ± 1.75, ± 2, ±2.25, ± 2.5, ± 2.75, ± 3, ± 3.25, ± 3.5, ± 3.75, ± 4, ± 4.25, ± 4.5, ± 4.75, ± 5, ± 5.5, ± 6, ± 6.5, ± 7, ± 7.5, ± 10, ± 15, ± 20, ± 25, ± 30, ± 100 and +300 parts per million (ppm).The scan duration was 5 minutes and 25 seconds.

### Image analysis

The tumor region-of-interest (ROI) was manually delineated by two dedicated radiologists (with 3 and 10 years of neuroradiology experience, respectively) on CEST images. Areas with necrosis, cysts, and hemorrhages were carefully excluded. The solid tumor was defined as either the contrast-enhanced region on T1-weighted images or the hyperintense region on T2-FLAIR images (when contrast enhancement was not detected) (25, 28, 29). Based on a previous study (30), we used custom MATLAB code (version 2023b, MathWorks, Natick, MA, USA) for quantitative image analysis of CEST. The Z-spectrum is generated using the ratio between the saturated image Ssat and the fully relaxed image S0. The spline-interpolated Z-spectrum was used to calculate frequency differences for generating B0 maps and performing voxel-wise B0 field correction. The B0-corrected Z-spectrum was then fitted as a sum of five Lorentzian functions corresponding to aliphatic nuclear Overhauser effect (NOE, -3.5 ppm), magnetization transfer (MT, -1 ppm), direct saturation of water (DS, 0 ppm), amine (2.0 ppm), and amide (3.5 ppm).

The spectrally selective CEST effects were obtained through Lorentzian line fitting for four steps: (1) motion correction using a subpixel image registration algorithms and denoising raw images using multilinear singular value decomposition; (2) 2-pool Lorentzian fitting (MT and DS) for B0 determination and B0 correction (3) 2-pool Lorentzian fitting (MT and DS) on B0 corrected data, generation of MTRLD; (4) 3-pool Lorentzian model fitting of MTRLD for isolated CEST contrast.

The first step involved utilizing a 2-pool model to characterize background signals such as direct water saturation (DS) and semisolid magnetization transfer (MT). Only those irradiation frequency offsets, assumed to be influenced exclusively by the background signal, were employed for the fit (MT: ± 10, ± 15, ± 20, ± 25, ± 30, ± 100; water: ± 1, ± 0.75, ± 0.5, ± 0.25, 0 ppm). Any other irradiation frequency offsets were disregarded. The 2-pool fit model used is expressed by the DS(w) and MT.


(1) $$Z\left(\text{Δ}\omega \right)=c-L_{\omega }-L_{MT}$$

with a constant c and the adjusted Lorentzian Lw of the water line. Lw includes a plateau to account for the pulse bandwidth at 3T defined by Equation 2.


(2) $$L_{\omega }=A_{\omega }\frac{\frac{{\text{Γ}}_{\omega }^{2}}{4}}{{\text{Γ}}_{\omega }^{2}/4+{\left(x\cdot \theta \left[x\right]+y\cdot \theta \left[-y\right]\right)}^{2}}$$

where A represents the Lorentzian amplitude of the five pools, Γ represents the Lorentzian width (full-width-at-half-maximum) of the five pools, and δ represents the peak position. Here, Θ[•] refers to the Heaviside function, with $x=\left(\text{Δ}\omega -\delta_{\omega }-\frac{BW}{2}\right)$ and $y=\left(\text{Δ}\omega -\delta_{\omega }+\frac{BW}{2}\right)$ . The parameter BW is an estimate of the Fourier width of the Gaussian saturation pulse, which is related to platform width and remains constant for $BW=\frac{1}{t_{pulse}\frac{\gamma }{2\pi }}$ . The second pool in which the Lorentzian function is defined in Equation 3 represents MT:


(3) $$L_{MT}=A_{MT}\frac{{\text{Γ}}_{MT}^{2}/4}{{\text{Γ}}_{MT}^{2}/4+{\left(\text{Δ}\omega -\delta_{MT}\right)}^{2}}$$

The Lorentzian ssMT pool was fitted with an initial resonance frequency of -1 ppm, which was adjustable within the range from 0 to -2.5 ppm during data fitting (30) In the second step, the water pool’s off-resonance in the preliminary 2-pool model served as a surrogate B0 map. Z-spectra underwent shifts to compensate B0 inhomogeneity.

In accordance with prior research, the Lorentzian difference method was employed for the evaluation of peak-selective CEST.


(4) $$MTR_{LD}=Z_{fit,ref}-Z$$

In step 3, the $Z_{fit,ref}$ referred to a 2-pool background fit, which was repeated on B0-corrected and denoised Z-spectra.

Ultimately, in the step 4, a 3-pool Lorentzian model was implemented to fit the *MTR_LD_* spectrum to distinctly separate the amide (+3.5 ppm), amine (+2.0 ppm), and NOE (-3.5 ppm) resonances.


(5) $$MTR_{LD}\left(\text{Δ}\omega \right)=c+L_{+2ppm}+L_{+3.5ppm}+L_{-3.5ppm.}$$

and (Equation 6)


(6) $$\;L_{x}=A_{x}\frac{\frac{{\text{Γ}}_{x}^{2}}{4}}{{\text{Γ}}_{x}^{2}/4+{\left(\text{Δ}\omega -\delta_{x}\right)}^{2}}$$

Quantitative maps were derived from the fitting parameter Ax for the five CEST pools.

The conventional magnetization transfer ratio (MTR) asymmetry analysis was used to calculate the MTR_3.5_, defined as


(7) $$\text{MT}{\text{R}}_{3.5}=\;\left(\text{Z}\left(-3.5\text{ ppm}\right)\;–\text{ Z}\left(+3.5\text{ ppm}\right)\right)/\text{M}0$$

We use CEST to characterize the acidity of the tumor region according to (17).


(8) $${\text{MTR}}_{\text{asym}}@3.0\text{ppm}\left(\text{pH}\right)=\text{α}+\frac{\text{β}-\text{α}}{1+10^{\text{δ}\left(\text{κ}\cdot \text{pH}\right)}}$$

At last, the histogram values for various parameters such as amide, NOE, amine, MT, DS, MTR_3.5_, and pH_weighted (13, 17) in tumor were calculated.

### Statistical analysis

Data were analyzed using SPSS 25.0, GraphPad Prism version 8.0, and MedCalc 20.0. The inter-observer variability of measurements in glioma patients was assessed using the intra-class correlation coefficient. Continuous variables with normal distribution were expressed as the mean ± SD, while non-normally distributed variables were expressed as the median with IQR. Categorical variables were expressed as frequencies. Metrics with significant differences were identified using an independent sample t-test (for normally distributed data) or Mann–Whitney U test (for non-normally distributed data). The Chi-squared test was used for categorical variables. Among the histogram features of amide, NOE, amine, MT, DS, pH_weighted, and MTR3.5, those with statistical significance (p < 0.05) were first selected. Features demonstrating the highest diagnostic performance were further selected. Collinearity analysis was performed on these features, and those with a tolerance (Tol) less than 0.1 or a variance inflation factor (VIF) greater than 10 were excluded. The remaining features were retained for constructing the combined model. Individual features or combined models were used for glioma grading and molecular typing (IDH mutation, 1p/19q codeletion, and MGMT promoter methylation status). The significance level was set at p = 0.05 for all tests.

## Results

### Patient information

The demographic and pathological findings of the participants are summarized in **Table 1**. A total of 128 patients with histologically confirmed gliomas were included. There were 24 grade II, 16 grade III, and 88 grade IV gliomas, among which 82 were IDH-wt and 46 were IDH-mut. Significant differences were found in age across different grades (p = 0.018) and IDH subtypes (p = 0.014). Among grade II and III gliomas, there were 18 and 22 with 1p/19q codeletion, respectively, and the remaining without codeletion. No significant differences in age were observed between these groups (p = 0.667, 0.683, respectively). There were 17 gliomas with MGMT promoter methylation and 16 without. Patients with MGMT promoter methylation were significantly older than those without (p = 0.043). No significant differences were found in gender across all subgroups (p = 0.785, 0.963, 0.214, 0.315, 0.728, respectively). We performed an inter-observer consistency analysis for all gliomas, low-grade gliomas, and high-grade gliomas separately, and the results showed good consistency in both groups. The intraclass correlation coefficients for inter-observer agreement for CEST metric values ranged from 0.90 to 0.99(**Supplementary Tables S1**–**S3**).

**Table 1:** Demographic information and pathological features of participants.

	Subtype	Number	Age (years)	*p*	Gender (male)	*p*
Tumor grade	II III IV	24 16 88	48 ± 10 45 ± 14 54 ± 14	0.018	24 (12) 16 (7) 88 (51)	0.785
IDH mutation	IDH-wt IDH-mut	82 46	54 ± 14 48 ± 12	0.014	82 (46) 46 (26)	0.963
1p/19q within grade II	codeletion noncodeletion	10 14	46 ± 10 48 ± 10	0.667	10 (7) 14 (5)	0.214
1p/19q within grade III	codeletion noncodeletion	8 8	45 ± 15 44 ± 14	0.813	8 (3) 8 (6)	0.315
MGMT promoter	methylation unmethylation	17 16	54 ± 11 46 ± 12	0.043	17 (11) 16 (9)	0.728

IDH, isocitrate dehydrogenase; IDH-wt, IDH wild type; IDH-mut, IDH mutant type; 1p/19q, chromosoe 1 and the long arm of chromosome 19; MGMT, O-6-methylguanine-DNA methyltransferase. The age is expressed as mean ± standard deviation.

### CEST metrics in distinguishing grade II and grade III gliomas

**Figure 2:**
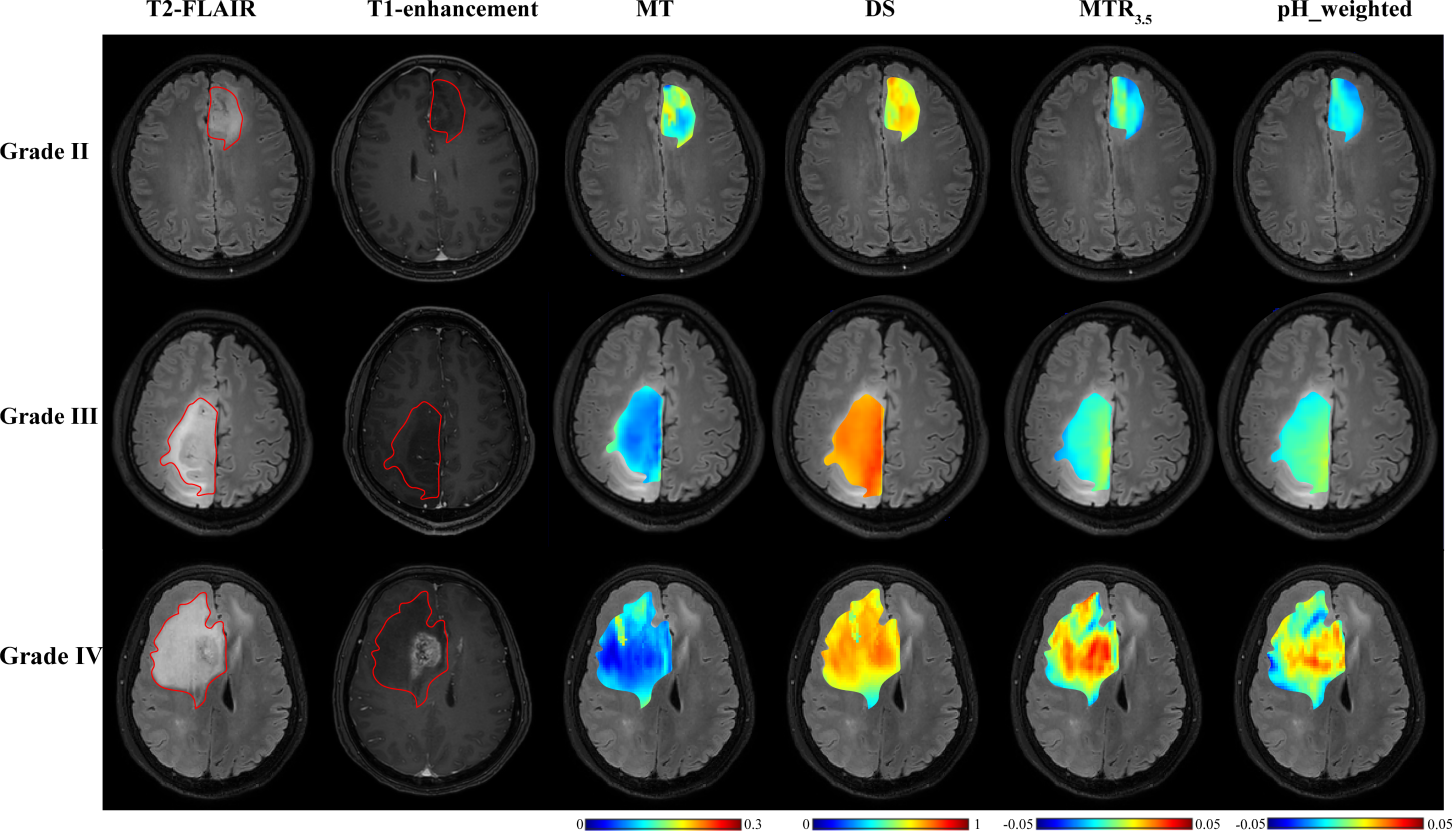
T2-FLAIR, T1-enhancement images and effective CEST derived metric maps of MT, DS, MTR3.5 and pH_weighted for differentiating between one grade II glioma patient (44 years old male), one grade III glioma patient (33 years old male) and one grade IV glioma patient (61 years old male). As glioma grade increases, tumors exhibit higher DS and MTR3.5 signals, lower MT signals, and increased acidity within the tumor region.

**Figure 3:**
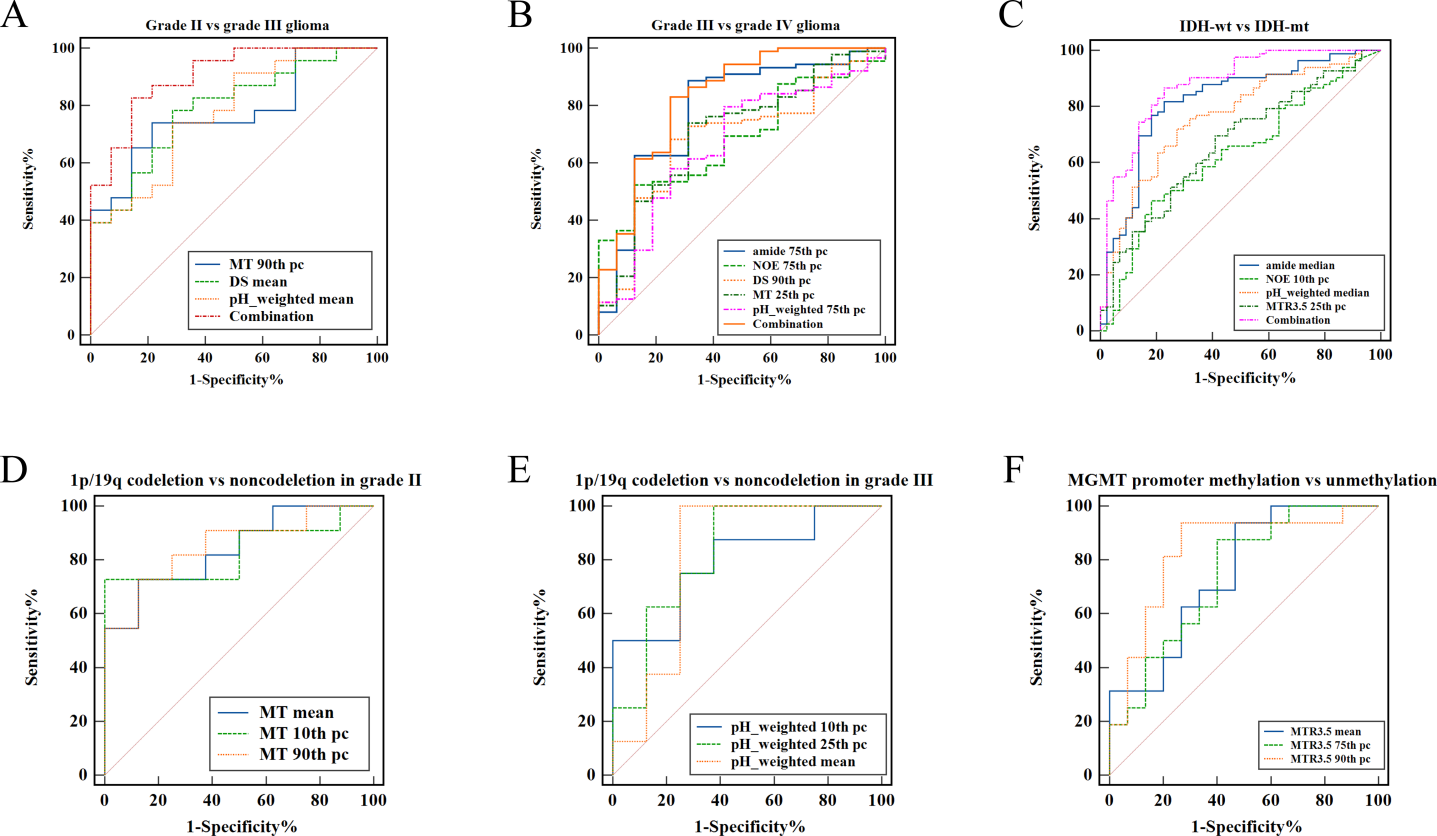
ROC curve analysis of fitted CEST metric histogram features in differentiating glioma subtype. **(A)** Differentiating grade II from grade III. **(B)** Differentiating grade III from grade IV. **(C)** Differentiating IDH-wt from IDH-mut. **(D)** Differentiating 1p/19q codeletion from 1p/19q non-codeletion in grade II glioma. **(E)** Differentiating 1p/19q codeletion from 1p/19q non-codeletion in grade III glioma. **(F)** Differentiating MGMT promoter methylation from unmethylation.

As shown in **Table 2** and **Figure 2**, grade III gliomas exhibited higher DS (median: 0.80 vs 0.78, p = 0.020) and pH_weighted (median: -0.01 vs -0.02, p = 0.008) signals, and lower MT (mean: 0.14 vs 0.15, p = 0.029) (**Supplementary Figure S1A**) compared to grade II gliomas. Specifically, the 90th percentile of MT [p = 0.003, AUC = 0.78 (95% CI: 0.62- 0.90)], the mean of DS [p = 0.020, AUC = 0.72 (95% CI: 0.55-0.85)], and the mean of pH_weighted [p = 0.006, AUC = 0.76 (95% CI: 0.59-0.88)] showed the best performance for each signal, respectively (**Figure 3A**, **Table 3**). The combined model achieved an AUC of 0.80 (95% CI: 0.64-0.91) (**Supplementary Table S4**).

**Table 2:** Results of histogram analyses of CEST for glioma grading.

CEST MRI MT mean	WHO II Median (Q1-Q3)	WHO III Median (Q1-Q3)	*p*
	0.15 (0.14-0.17)	0.14 (0.11-0.15)	0.029
DS mean	0.78 (0.76-0.79)	0.80 (0.78-0.84)	0.020
pH_weighted mean	-0.01 (-0.02- -0.01)	-0.01 (-0.01- -0.001)	0.006
DS median	0.78 (0.75-0.79)	0.80 (0.78-0.84)	0.023
pH_weighted median	-0.01 (-0.02- -0.01)	-0.01 (-0.01- -0.002)	0.008
DS 10th pc	0.74 (0.70-0.75)	0.76 (0.73-0.78)	0.021
pH_weighted 10th pc	-0.03 (-0.04- -0.02)	-0.02 (-0.02-0.02)	0.025
DS 25th pc	0.76 (0.73-0.77)	0.78 (0.76-0.81)	0.025
pH_weighted 25th pc	-0.02 (-0.03- -0.01)	-0.01 (-0.02- -0.01)	0.007
MT 75th pc	0.18 (0.16-0.21)	0.16 (0.13-0.18)	0.033
DS 75th pc	0.80 (0.78-0.82)	0.82 (0.80-0.86)	0.029
pH_weighted 75th pc	-0.01 (-0.01- -0.001)	-0.001 (-0.01- 0.01)	0.020
MT 90th pc	0.20 (0.17-0.24)	0.17 (0.14-0.18)	0.003

amide mean	WHO III Median (Q1-Q3)	WHO IV Median (Q1-Q3)	*p*
	0.04 (0.04-0.05)	0.06 (0.05-0.06)	0.001
NOE mean	0.06 (0.05-0.06)	0.06 (0.05-0.07)	0.034
MT mean	0.14 (0.11-0.15)	0.16 (0.13-0.18)	0.031
DS mean	0.80 (0.78-0.84)	0.78 (0.75-0.80)	0.013
amide median	0.05 (0.04-0.05)	0.06 (0.05-0.06)	0.001
MT median	0.14 (0.11-0.15)	0.16 (0.13-0.18)	0.033
DS median	0.80 (0.78-0.84)	0.78 (0.75-0.80)	0.012
DS 10th pc	0.76 (0.74-0.78)	0.73 (0.70-0.75)	0.015
amide 25th pc	0.04 (0.03-0.04)	0.05 (0.04-0.06)	0.005
DS 25th pc	0.78 (0.76-0.81)	0.75 (0.73-0.77)	0.009
amide 75th pc	0.05 (0.05-0.06)	0.06 (0.06-0.07)	<0.001
NOE 75th pc	0.07 (0.06-0.07)	0.07 (0.06-0.08)	0.017
MT 75th pc	0.16 (0.13-0.18)	0.18 (0.15-0.21)	0.017
DS 75th pc	0.82 (0.80-0.86)	0.80 (0.77-0.83)	0.020
pH_weighted 75th pc	-0.003 (-0.007-0.008)	0.008 (0.001-0.016)	0.037
amide 90th pc	0.06 (0.05-0.07)	0.07 (0.06-0.08)	0.001
NOE 90th pc	0.07 (0.06-0.07)	0.08 (0.07-0.08)	0.030
MT 90th pc	0.17 (0.15-0.20)	0.20 (0.17-0.23)	0.018

The CEST histogram features for effectively grading gliomas are expressed as median (Q1-Q3). pc, percentile.

**Table 3:** The diagnostic performance of signals in evaluating tumor grades, 1p/19q codeletion status and MGMT promoter methylation status.

	CEST MRI	Cutoff	Sensitivity	Specificity	AUC
Grade II vs grade III glioma	MT mean	0.14	83.33% (20/24)	56.25% (9/16)	0.70 (0.53-0.83)
	DS mean	0.79	79.17% (19/24)	62.50% (10/16)	0.72 (0.55-0.85)
	pH_weighted mean	-0.01	75.00% (18/24)	68.75% (11/16)	0.76 (0.59-0.88)
	DS median	0.79	79.17% (19/24)	62.50% (10/16)	0.71 (0.55-0.85)
	pH_weighted median	-0.01	54.17% (13/24)	93.75% (15/16)	0.76 (0.60-0.88)
	DS 10th pc	0.74	75.00% (18/24)	68.75% (11/16)	0.72 (0.55-0.85)
	ph_weighted 10th pc	-0.03	62.50% (15/24)	87.50% (14/16)	0.71 (0.55-0.85)
	DS 25th pc	0.77	79.17% (19/24)	62.50% (10/16)	0.71 (0.55-0.84)
	pH_weighted 25th pc	-0.02	54.17% (13/24)	93.75% (15/16)	0.76 (0.60-0.88)
	MT 75th pc	0.16	66.67% (16/24)	75.00% (12/16)	0.70 (0.54-0.84)
	DS 75th pc	0.79	50.00% (12/24)	87.50% (14/16)	0.71 (0.54-0.84)
	pH_weighted 75th pc	-0.008	54.17% (13/24)	81.25% (13/16)	0.73 (0.57-0.86)
	MT 90th pc	0.18	75.00% (18/24)	78.57% (11/14)	0.78 (0.62-0.90)
	Combination		62.50% (15/24)	93.75% (15/16)	0.80 (0.64-0.91)
Grade III vs grade IV glioma	amide mean	0.04	90.91% (80/88)	62.50% (10/16)	0.77 (0.68-0.85)
	NOE mean	0.06	60.23% (53/88)	75.00% (12/16)	0.67 (0.57-0.76)
	MT mean	0.15	54.55% (48/88)	81.25% (13/16)	0.67 (0.57-0.76)
	DS mean	0.77	47.73% (42/88)	87.50% (14/16)	0.70 (0.60-0.78)
	amide median	0.05	76.14% (67/88)	75.00% (12/16)	0.76 (0.67-0.84)
	MT median	0.15	56.82% (50/88)	81.25% (13/16)	0.67 (0.57-0.76)
	DS median	0.78	50.00% (44/88)	87.50% (14/16)	0.70 (0.60-0.78)
	DS 10th pc	0.76	77.27% (68/88)	62.50% (10/16)	0.69 (0.59-0.78)
	amide 25th pc	0.04	63.64% (56/88)	81.25% (13/16)	0.72 (0.62-0.80)
	DS 25th pc	0.77	73.86% (65/88)	68.75% (11/16)	0.71 (0.61-0.79)
	amide 75th pc	0.05	88.64% (78/88)	68.75% (11/16)	0.78 (0.69-0.86)
	NOE 75th pc	0.07	52.27% (46/88)	87.50% (14/16)	0.69 (0.59-0.78)
	MT 75th pc	0.16	68.18% (60/88)	75.00% (12/16)	0.69 (0.59-0.78)
	DS 75th pc	0.79	44.32% (39/88)	93.75% (15/16)	0.68 (0.59-0.77)
	pH_weighted 75th pc	0.001	79.55% (70/88)	56.25% (9/16)	0.67 (0.57-0.75)
	amide 90th pc	0.06	89.77% (79/88)	56.25% (9/16)	0.75 (0.66-0.83)
	NOE 90th pc	0.08	51.14% (45/88)	100.00% (16/16)	0.67 (0.57-0.76)
	MT 90th pc	0.18	68.18% (60/88)	75.00% (12/16)	0.69 (0.59-0.77)
	Combination		89.77% (79/88)	75.00% (12/16)	0.83 (0.74-0.90)
IDH-wt vs IDH-mt	amide mean	0.05	82.93% (68/82)	69.57% (32/46)	0.80 (0.72-0.87)
	NOE mean	0.05	85.37% (70/82)	41.30% (19/46)	0.62 (0.53-0.70)
	pH_weighted mean	-0.004	62.20% (51/82)	71.74% (33/46)	0.69 (0.61-0.77)
	MTR_3.5_ mean	-0.01	75.61% (62/82)	52.17% (24/46)	0.64 (0.56-0.73)
	amide median	0.05	81.71% (67/82)	78.26% (36/46)	0.83 (0.75-0.89)
	pH_weighted median	-0.006	71.95% (59/82)	71.74% (33/46)	0.76 (0.68-0.83)
	MTR_3.5_ median	-0.01	81.71% (67/82)	45.65% (21/46)	0.66 (0.57-0.74)
	amide 10th pc	0.03	56.10% (46/82)	84.78% (39/46)	0.70 (0.61-0.78)
	NOE 10th pc	0.05	46.34% (38/82)	82.61% (38/46)	0.64 (0.55-0.72)
	pH_weighted 10th pc	-0.02	53.66% (44/82)	80.43% (37/46)	0.66 (0.57-0.74)
	MTR_3.5_ 10th pc	-0.02	42.68% (35/82)	82.61% (38/46)	0.64 (0.55-0.73)
	amide 25th pc	0.04	86.59% (71/82)	69.57% (32/46)	0.80 (0.72-0.86)
	NOE 25th pc	0.05	70.73% (58/82)	60.87% (28/46)	0.63 (0.54-0.72)
	pH_weighted 25th pc	-0.01	54.88% (45/82)	84.78% (39/46)	0.72 (0.63-0.80)
	MTR_3.5_ 25th pc	-0.02	69.51% (57/82)	58.70% (27/46)	0.67 (0.58-0.75)
	amide 75th pc	0.05	86.59% (71/82)	67.39% (31/46)	0.80 (0.72-0.87)
	pH_weighted 75th pc	0.002	71.95% (59/82)	71.74% (33/46)	0.74 (0.66-0.82)
	MTR_3.5_ 75th pc	-0.001	65.85% (54/82)	58.70% (27/46)	0.62 (0.53-0.71)
	amide 90th pc	0.06	89.02% (73/82)	58.70% (27/46)	0.74 (0.66-0.82)
	pH_weighted 90th pc	0.01	73.17% (60/82)	65.22% (30/46)	0.70 (0.61-0.77)
	Combination		84.15% (69/82)	82.61% (38/46)	0.84 (0.77-0.90)
1p/19q codeletion status within grade II glioma	MT mean	0.15	75.00% (9/12)	87.50% (5/8)	0.85 (0.63-0.97)
	MT 10th pc	0.11	75.00% (9/12)	88.89% (8/9)	0.81 (0.58-0.94)
	MT 25th pc	0.13	66.67% (8/12)	88.89% (8/9)	0.78 (0.55-0.93)
	MT 90th pc	0.20	75.00% (9/12)	88.89% (8/9)	0.87 (0.65-0.98)
1p/19q codeletion status within grade III glioma	pH_weighted mean	-0.01	100.00% (8/8)	75.00% (6/8)	0.81 (0.54-0.96)
	pH_weighted10th pc	-0.02	87.50% (7/8)	62.50% (5/8)	0.80 (0.53-0.95)
	pH_weighted 25th pc	-0.02	100.00% (8/8)	62.50% (5/8)	0.83 (0.56-0.97)
MGMT promoter methylation status	MTR_3.5_ mean	0.01	88.24% (15/17)	50.00% (8/16)	0.70 (0.52-0.85)
	MTR_3.5_ 75th pc	0.01	87.50% (14/16)	56.25% (9/16)	0.71 (0.52-0.86)
	MTR_3.5_ 90th pc	0.02	88.24% (15/17)	73.33% (11/15)	0.79 (0.61-0.91)

Using ROC curves to evaluate the efficacy of CEST histogram features in grading gliomas, identifying 1p19q codeletion and MGMT promoter methylation status.

### CEST metrics in distinguishing grade III and grade IV gliomas

As shown in **Table 2** and **Figure 2**, grade IV gliomas exhibited higher amide (mean: 0.06 vs 0.04, p = 0.001) (**Supplementary Figure S1B**), NOE (mean: 0.06 vs 0.05, p = 0.034), MT (mean: 0.16 vs 0.14, p = 0.031), pH_weighted (75th pc: 0.008 vs -0.003, p = 0.037), and lower DS (mean: 0.78 vs 0.80, p = 0.013) compared to grade III gliomas. Specifically, the 75th percentile of amide [p < 0.001, AUC = 0.78 (95% CI: 0.69-0.86)], the 75th percentile of NOE [p = 0.017, AUC = 0.69 (95% CI: 0.59-0.78)], the 90th percentile of MT [p = 0.018, AUC = 0.69 (95% CI: 0.59-0.78)], the 25th percentile of DS [p = 0.009, AUC = 0.71 (95% CI: 0.61-0.79)], and the 75th percentile of pH_weighted [p = 0.037, AUC = 0.67 (95% CI: 0.57-0.75)] showed the best performance for each signal, respectively (**Figure 3B**, **Table 3**). The combined model achieved an AUC of 0.83 (95% CI: 0.74-0.90) (**Supplementary Table S4**).

### CEST metrics in distinguishing IDH wide type and mutant type gliomas

**Figure 4:**
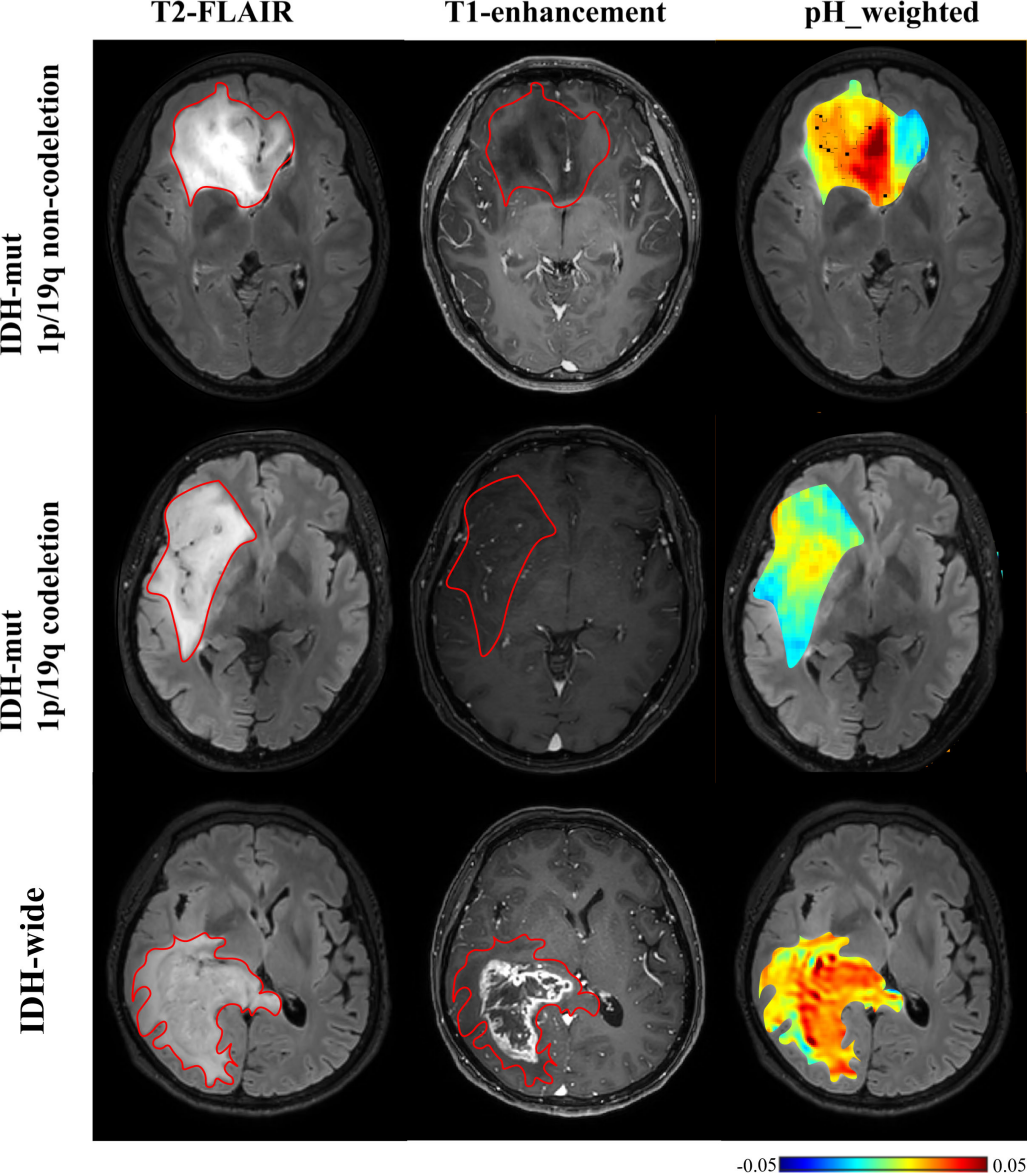
T2-FLAIR, T1-enhancement images and effective CEST derived metric maps of pH_weighted for differentiating between one IDH-mt with 1p/19q noncodeletion glioma patient (29 years old female, WHO III), one IDH- mt with 1p/19q noncodeletion glioma patient (60 years old female, WHO III) and one IDH wide glioma patient(66 years old female, WHO IV). IDH-wt manifested higher tumor acidity compared to IDH-mut. The glioma with 1p/19q codeletion appears to show lower acidity compared to the glioma with no 1p/19q codeletion.

As shown in **Table 4**, **Figure 4**, IDH-wt gliomas exhibited higher amide (mean: 0.06 vs 0.04, p < 0.001) (**Supplementary Figure S1E**), NOE (mean: 0.06 vs 0.07, p = 0.031), pH_weighted (mean: -0.002 vs -0.009, p < 0.001), and MTR3.5 (mean: 0.003 vs -0.010, p = 0.009) compared to IDH-mut gliomas. Specifically, the median of amide [p < 0.001, AUC = 0.83 (95% CI: 0.75-0.89)], the 10th percentile of NOE [p = 0.013, AUC = 0.64 (95% CI: 0.55-0.72)], the median of pH_weighted [p < 0.001, AUC = 0.76 (95% CI: 0.68-0.83)] and the 25th percentile of MTR3.5 [p = 0.002, AUC = 0.67 (95% CI: 0.58-0.75)] performed the best for each type of signal, respectively (**Figure 3C**, **Table 3**). The combined model achieved an AUC of 0.84 (95% CI: 0.77-0.90) (**Supplementary Table S4**).

**Table 4:** Results of histogram analyses of CEST for distinguishing between IDH-wt and IDH-mut gliomas.

CEST MRI	IDH-mut Median (Q1-Q3)	IDH-wt Median (Q1-Q3)	P
amide mean	0.04 (0.04-0.05)	0.06 (0.05-0.06)	<0.001
NOE mean	0.06 (0.05-0.06)	0.06 (0.05-0.07)	0.031
pH_weighted mean	-0.009 (-0.018- -0.004)	-0.002 (-0.008- 0.007)	<0.001
MTR_3.5_ mean	-0.010 (-0.019- -0.002)	-0.003 (-0.010-0.003)	0.009
amide median	0.04 (0.04-0.05)	0.06 (0.05-0.06)	<0.001
pH_weighted median	-0.01 (-0.017- -0.004)	-0.004 (-0.007-0.006)	<0.001
MTR_3.5_ median	0.10 (0.07-0.14)	0.16 (0.13-0.19)	<0.001
amide 10th pc	0.03 (0.02-0.03)	0.04 (0.03-0.05)	<0.001
NOE 10th pc	0.04 (0.03-0.04)	0.04 (0.04-0.05)	0.013
pH_weighted 10th pc	-0.03 (-0.03- -0.02)	-0.02 (-0.03- -0.01)	0.002
MTR_3.5_ 10th pc	-0.03 (-0.04- -0.02)	-0.02 (-0.03- -0.01)	0.008
amide 25th pc	0.04 (0.03-0.04)	0.05 (0.04-0.06)	<0.001
NOE 25th pc	0.05 (0.04-0.06)	0.05 (0.05-0.06)	0.020
pH_weighted 25th pc	-0.02 (-0.02- -0.01)	-0.01 (-0.02- -0.01)	<0.001
MTR_3.5_ 25th pc	-0.02 (-0.03- -0.01)	-0.01 (-0.02- -0.01)	0.002
amide 75th pc	0.05 (0.04-0.06)	0.06 (0.06-0.07)	<0.001
pH_weighted 75th pc	-0.002 (-0.011- 0.004)	0.008 (0.001-0.016)	<0.001
MTR_3.5_ 75th pc	-0.002 (-0.010- 0.006)	0.004 (-0.005- 0.129)	0.027
amide 90th pc	0.06 (0.05-0.07)	0.07 (0.06-0.08)	<0.001
pH_weighted 90th pc	0.004 (-0.006-0.017)	0.013 (0.006-0.026)	<0.001

The CEST histogram features for effectively distinguishing between IDH-wt and IDH-mt gliomas are expressed as median (Q1-Q3).

### CEST metrics in detecting 1p19q codeletion status

**Figure 5:**
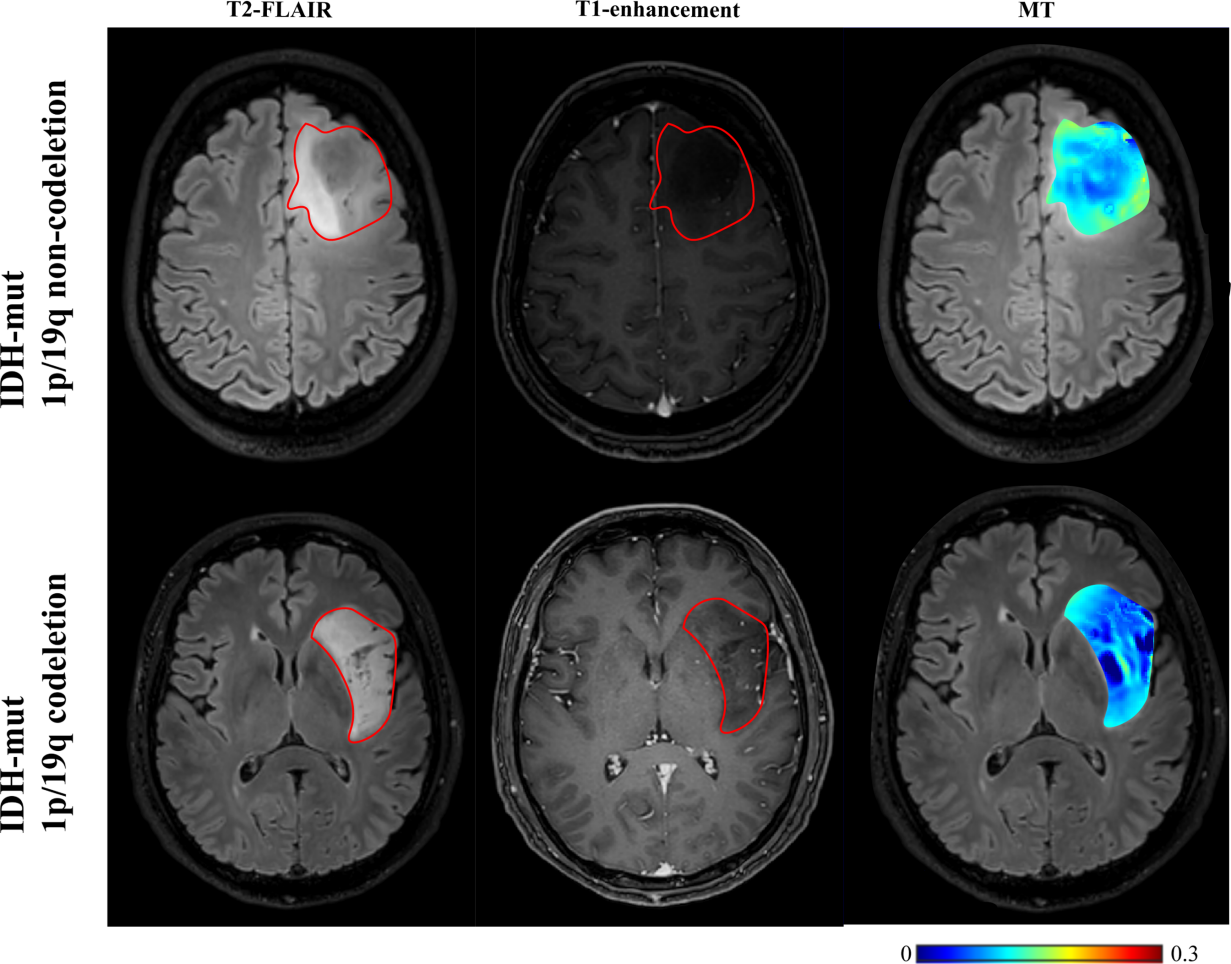
T2-FLAIR, T1-enhancement images and effective CEST derived metric maps of MT for differentiating between one IDH-mt with 1p/19q noncodeletion glioma (45 years old female, WHO II), one IDH-mt with 1p/19q codeletion glioma patient (57 years old male, WHO II). The glioma with 1p/19q codeletion appears to show lower MT compared to the glioma with no 1p/19q codeletion.

As shown in **Table 5** and **Figures 4**, **5**, for grade II gliomas, 1p/19q non-codeletion gliomas exhibited higher MT compared to 1p/19q codeletion gliomas (mean: 0.17 vs 0.14, p = 0.007) (**Supplementary Figure S1C**). The 90th percentile of MT achieved the best performance [p = 0.003, AUC = 0.87 (95% CI: 0.65-0.98)] (**Figure 3D**, **Table 3**). For grade III gliomas, 1p/19q non-codeletion gliomas exhibited higher pH_weighted signals compared to 1p/19q codeletion gliomas (mean: -0.004 vs -0.013, p = 0.038) (**Supplementary Figure S1D**). The 25th percentile of pH_weighted achieved the best performance [p = 0.028, AUC = 0.83 (95% CI: 0.56-0.97)] (**Figure 3E**, **Table 3**).

**Table 5:** Results of histogram analyses of CEST for identifying 1p/19q codeletion and MGMT promoter methylation.

	CEST metrics	1p/19q codeletion median (Q1-Q3)	1p/19q non-codeletion median (Q1-Q3)	*p*
Grade II	MT mean MT 10th pc MT 25th pc MT 90th pc	0.14 (0.12-0.15) 0.11 (0.08-0.11) 0.12 (0.11-0.13) 0.18 (0.16-0.19)	0.17 (0.15-0.17) 0.12 (0.10-0.15) 0.14 (0.12-0.16) 0.22 (0.20-0.25)	0.007 0.018 0.034 0.003
Grade III	pH_weighted mean pH_weighted10th pc pH_weighted 25th pc	-0.013 (-0.015- -0.003) -0.024 (-0.028- -0.020) -0.017 (-0.021- -0.012)	-0.004 (-0.005-0.002) -0.017 (-0.023- -0.011) -0.010 (-0.013- -0.007)	0.038 0.049 0.028
		Methylation	Unmethylation	
MGMT	MTR_3.5_ mean MTR_3.5_ 75th pc MTR_3.5_ 90th pc	-0.01 (-0.02-0.01) 0.01 (-0.01-0.01) 0.01 (0.01-0.02)	0.01 (-0.01-0.01) 0.02 (0.01-0.03) 0.02 (0.02-0.05)	0.048 0.043 0.005

The CEST histogram features for effectively identifying 1p/19q codeletion within grade II, III, respectively and MGMT promoter methylation status. MTR3.5, magnetization transfer ratio at 3.5 parts per million (ppm).

### CEST metrics in detecting MGMT promoter methylation status

**Figure 6:**
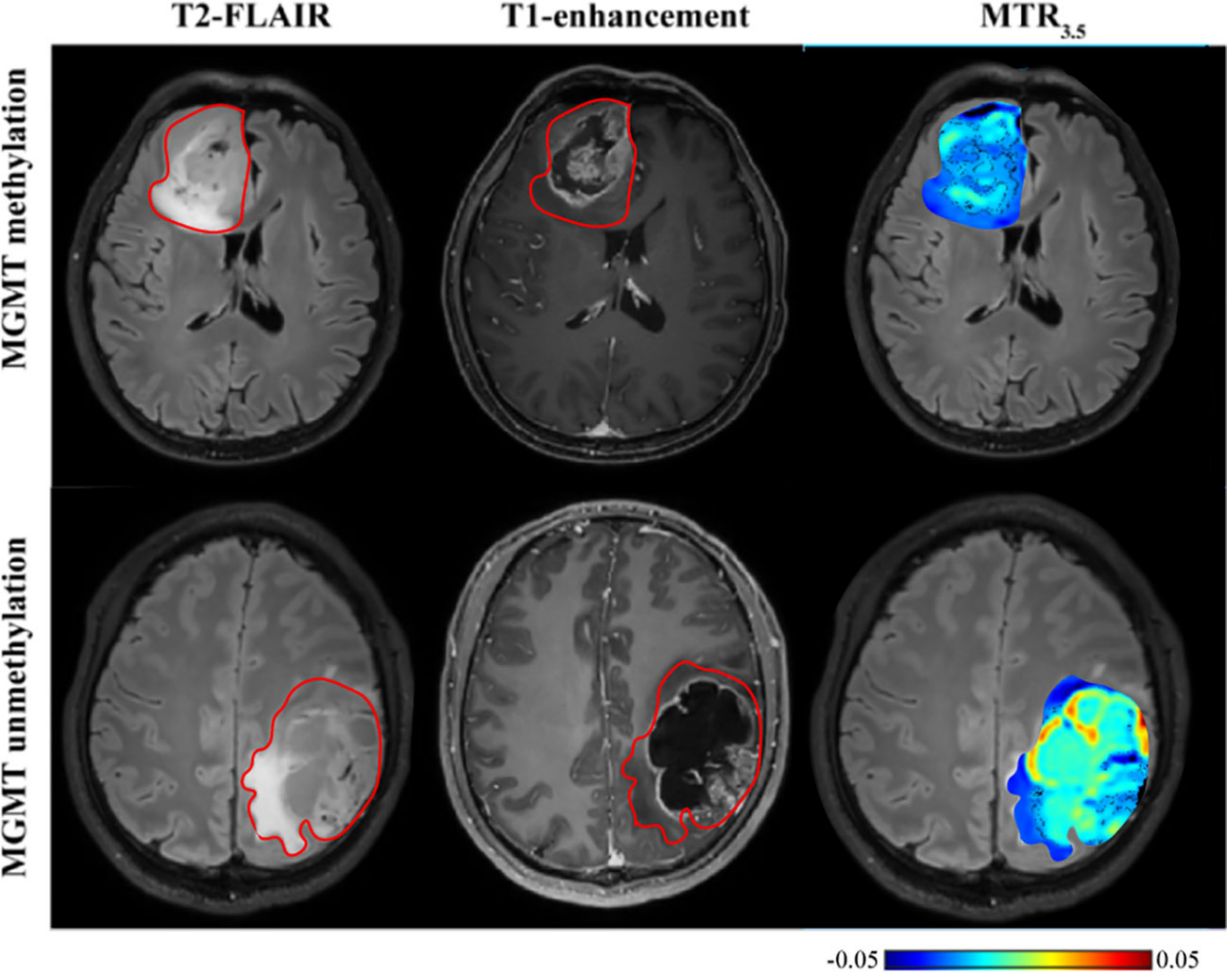
T2-FLAIR, T1-enhancement images and effective CEST derived metric map of MTR_3.5_ for differentiating between one MGMT promoter methylation glioma (53 years old female, WHO IV) and one MGMT promoter unmethylation glioma patient (30 years old male, WHO IV). MGMT promoter methylation glioma manifested higher MTR_3.5_ compared to unmethylation glioma.

As shown in **Table 5** and **Figure 6**, MGMT promoter unmethylated gliomas exhibited higher MTR3.5 signals compared to MGMT promoter methylated gliomas (mean: 0.01 vs -0.01, p = 0.048) (**Supplementary Figure S1F**). The 90th percentile of MTR3.5 achieved the best performance [p = 0.005, AUC = 0.79 (95% CI: 0.61-0.91)] (**Figure 3F**, **Table 3**).

## Discussion

This study investigated glioma grading and molecular genotyping using CEST-based pH assessment and micro- metabolic profiling within the context of the 2021 WHO CNS classification. Our results indicate that multi-pool Lorentzian analysis and pH-weighted analysis demonstrate diagnostic performance in grading gliomas and ingenotyping for IDH mutation status, 1p/19q co-deletion, and MGMT promoter methylation status.pH_weighted imaging can characterize the acidic microenvironment of tumors. We found that high-grade gliomas, IDH-wt gliomas, and 1p/19q non-codeleted gliomas exhibited higher pH_weighted values compared to low-grade gliomas, IDH-mt gliomas, and 1p/19q codeleted gliomas. Tumor cells preferentially convert glucose to lactic acid even in the presence of oxygen, resulting in excessive lactic acid production. Additionally, poor vascularization in these tumors leads to hypoxic conditions that further drive glycolysis and acid production (15). The higher metabolic activity in more invasive gliomas results in hypoxia and the accumulation of acidic metabolic products, leading to larger pH_weighted values (31). As shown in **Figure 5**, gliomas with and without 1p/19q codeletion are difficult to differentiate on T2-FLAIR and T1-enhancement images. However, pH_weighted imaging can visually highlight differences between them and better reflect tumor heterogeneity. In the central tumor region, acidity is significantly increased, while in the peritumoral edema zone, tumor acidity is relatively lower. In more invasive IDH-wt gliomas, both the central tumor region and the peritumoral edema zone exhibit higher acidity, partially explaining their greater invasiveness. Therefore, we believe that pH_weighted imaging is a promising biomarker for glioma grading and subtyping analysis.

MT and DS respectively represent the content of semi-solid molecular tissue and water molecules. Grade III gliomas exhibited higher DS and lower MT compared to grade II gliomas. DS is related to tissue water proton density. Research indicates that high-grade gliomas tend to have higher vascular endothelial growth factor (VEGF) expression (32). VEGF is known as a potent growth factor for vascular endothelial cells, playing a crucial role in tumor growth and invasion by promoting the proliferation and migration of tumor vascular endothelial cells, increasing tumor vascular permeability, and inducing tumor lymphangiogenesis (32, 33). The higher VEGF expression corresponds with more severe edema, resulting in higher DS (25). However, our findings showed that DS was lower in grade IV gliomas compared to grade III gliomas, possibly due to differences in ROI selection. In grade III gliomas, where most cases did not show enhancement on T1-weighted images, the entire T2 hyperintense region was selected as the ROI. In contrast, grade IV gliomas were characterized by selecting the enhanced T1 area and excluding the peritumoral edema zone. MT primarily originates from immobile macromolecules, such as proteins and polysaccharides, and may serve as an indicator of white matter integrity (34). MT was higher in grade II gliomas, likely due to their retention of more normal brain tissue structure and composition. In grade IV gliomas, elevated cell density may lead to increased levels of proteins, polysaccharides, and other components within the tumor region, resulting in a higher MT effect. Furthermore, MT was associated with 1p/19q codeletion in grade II gliomas, with 1p/19q codeletion gliomas showing lower MT (**Figure 4**). Further subclassification of 1p/19q codeletion and non-codeletion within low-grade gliomas is meaningful, as the identification of 1p/19q codeletion in IDH-mt gliomas maybe influenced by histological grading. Distinguishing 1p/19q subtypes in grade II/III gliomas can facilitate more precise treatment planning and efficacy assessment in preoperative or postoperative follow-up.

Amide and NOE reflect the content of amide protons and macromolecules such as lipids within the tissue. Significant differences were observed in amide and NOE signals between grade III and IV gliomas, as well as between IDH-wt and IDH-mt gliomas. IDH-wt gliomas are typically more aggressive and have higher cellular density compared to IDH-mt gliomas. This aggressive phenotype is associated with an increased proliferative rate and elevated protein synthesis (23, 35). In contrast, IDH-mt leads to the production of the oncometabolite 2- hydroxyglutarate (2-HG), which results in abnormal methylation of DNA and histones, affecting gene expression and cell differentiation (36). The higher concentration of proteins and peptides in IDH-wt gliomas likely contributes to a stronger amide and NOE signal. We found that the diagnostic performance of amide is superior to MTR_3.5_. This maybe because MTR_3.5_ is influenced by signals such as NOE and DS, and therefore cannot reflect a purer source of the amide signal.

Our study also found that MGMT promoter unmethylated gliomas typically exhibit higher amide signals. MGMT promoter methylation in gliomas is associated with reduced protein expression, which may impact the expression of downstream proteins. Therefore, CEST may serve as a useful imaging biomarker for predicting MGMT methylation status, consistent with previous findings (37). In contrast to previous studies where APT could not predict MGMT promoter methylation, possibly due to smaller sample sizes (26), our results suggest that MTR_3.5_, despite being affected by multiple factors, can predict MGMT promoter methylation more effectively than the relatively pure amide signals.

In our study, the amine signal showed no significant differences in the grading and molecular classification of gliomas. Notably, Zhu et al.’s research also found no differences in the amine signal between IDH wild-type and mutant gliomas (27). We believe that there are two possible reasons for this result. Firstly, the amine signal has been assumed to mainly represent the contribution from creatine amine protons. However, amine signal obtained through Lorentzian fitting frequently overlaps with other rapidly exchanging pools, like glutamate, making it difficult to isolate them under 3T conditions. Creatine provides phosphate through phospho-creatine for adenosine triphosphate synthesis in the cell energy requirement. Tumor has reduced creatine and tumor creatine further reduces with tumor progression presumably due to elevated energy deficiency (38). There is building evidence that alterations to glutamate homeostasis in gliomas play an important role in diffuse glioma cell survival and increased extra-cellular glutamate causes excitotoxicity to peri-tumoral structures and promotes tumor invasion in pre-clinical studies (39). The complex variations in the contents of various components within the tumor lead to fluctuations in the amine signal. Secondly, our study was conducted using a 3T MRI scanner. Due to the relatively fast exchange rate of the amine signal and its fitting being close to the water peak at 2 ppm, the z-spectrum characteristics may not be distinct enough, making the Lorentzian fitting more challenging. In summary, the amine signal in glioma research may be influenced by various factors. Further research may need to explore more sensitive techniques or methods to better understand the role of the amine signal in gliomas. Based on the above discussion, the combination of multi-pool Lorentz analysis and pH analysis based on CEST demonstrates good performance in improving the grading and IDH gene typing of glioma. MT and pH_weighted can effectively identify 1p/19q codeletion in grade II and grade III gliomas, respectively. MTR_3.5_ demonstrates potential effect in identifying MGMT promoter methylation. This technology can be implemented on standard MRI equipment, with a scanning time of approximately 5 minutes being clinically feasible. It does not require additional injection of contrast agents, making it relatively safe. In our study, we chose to perform the scans before the injection of the contrast agent to avoid the influence of the contrast agent on the CEST effect (40).

However, our study has several limitations. Firstly, although existing literature has demonstrated that, within lower irradiation power ranges, multi-pool Lorentzian fitting offers superior quantification accuracy compared to the three-frequency offset method and the Lorentzian-Dipolar (LD) method (38). However, multi-pool Lorentzian fitting has several limitations. In situations where the resonance frequencies of different signals are closely spaced or mixed, Lorentzian fitting may struggle to effectively differentiate between these signals. For example, the wide ‘MT’ peak could have multiple contributions especially the NOE(-1.6), which have attracted many interests in recent years (41–44). However, we could not resolve these components precisely in our analysis. Such spectral overlap can lead to inaccuracies in the fitting results, adversely impacting the quantification of specific signals, such as those from amines or other metabolites (27). Besides, Lorentzian fitting exhibits high sensitivity to background noise, particularly when signal intensities are low. The presence of background noise can interfere with the fitting process, resulting in erroneous parameter estimates. Additionally, successful Lorentzian fitting requires careful selection of initial parameters and fitting ranges. Inappropriate parameter choices can lead to convergence on local minima, thus compromising the accuracy and reliability of the fitting results. These limitations underscore the importance of judiciously selecting appropriate fitting methods and parameters in practical applications to ensure the reliability and validity of the results. Secondly, for pH assessment, although research indicates that amine proton-based CEST imaging (with a resonance frequency of approximately 3.0 ppm) can provide pH-weighted image contrast and may serve as an important imaging biomarker for human brain gliomas (17). The measured CEST contrast depends on various technical factors, including the shape, duration, length, amplitude and repetition time of the saturation pulse, and the strength of the scanning field, and the concentration of amine protons. Additionally, the image SNR can affect pH measurements (45). Furthermore, exchangeable protons from other proteins or macromolecules within the tissue may also influence the amine signal (27).Further research is needed to standardize CEST scanning protocols and post-processing techniques to optimize signal acquisition and data fitting. Additionally, larger-scale clinical studies are required to investigate pH variations among different tumor grades and molecular subtypes across the entire tumor. Thirdly, the sample size is relatively small, particularly for the 1p/19q expression status subgroup. Further validation in a larger cohort is necessary. As a single-center study, there are inherent limitations such as reduced generalizability and potential biases. Multi-center studies are needed to validate and expand upon these findings. Lastly, due to time constraints, only 2D single-slice imaging was performed, which might have missed important pathological regions due to intra-tumoral heterogeneity. Implementing 3D acquisition to cover the entire tumor could address this issue.

## Conclusion

In summary, our findings indicate that quantitative assessment of tumor metabolism and microenvironment acidity through multi-pool Lorentzian analysis and pH-weighted analysis can serve as indicators for glioma grading, and for predicting IDH mutations, 1p/19q codeletion, and MGMT promoter methylation status. These metrics not only provide valuable insights into tumor subgroups but also reflect the heterogeneity within tumors.

## Data Availability

The raw data supporting the conclusions of this article will be made available by the authors, without undue reservation.
