# Supplementary material for: Multipool-CEST and CEST-based pH assessment as predictive tools for glioma grading, IDH mutation, 1p/19q codeletion, and MGMT promoter methylation in gliomas
Source: Front Oncol. 2024 Dec 20;14:1507335. doi: 10.3389/fonc.2024.1507335 (PMC11695364; doi:10.3389/fonc.2024.1507335)
Supplement: Supplementary file 1 [file DataSheet1.docx]

**Supplementary information**

Table S1. Interobserver agreement for measurements of CEST metrics

|  | ICC (95CI%) | P |
| --- | --- | --- |
| amide | 0.896(0.848-0.929) | <0.001 |
| NOE | 0.900(0.855-0.932) | <0.001 |
| amine | 0.914(0.875-0.933) | <0.001 |
| MT | 0.937(0.907-0.951) | <0.001 |
| DS | 0.967(0.950-0.975) | <0.001 |
| pH | 0.991(0.986-0.994) | <0.001 |
| MTR_3.5_ | 0.938(0.910-0.958) | <0.001 |

ICC interclass correlation coefficient. Data in parentheses are 95% confidence intervals

Table S2. Interobserver agreement for measurements of CEST metrics in low grade gliomas

|  | ICC (95CI%) | P |
| --- | --- | --- |
| amide | 0.978(0.842-0.997) | <0.001 |
| NOE | 0.971(0.795-0.996) | <0.001 |
| amine | 0.969(0.779-0.996) | 0.001 |
| MT | 0.953(0.664-0.993) | 0.002 |
| DS | 0.982(0.868-0.997) | <0.001 |
| pH | 0.972(0.799-0.996) | 0.001 |
| MTR_3.5_ | 0.970(0.787-0.996) | 0.001 |

ICC interclass correlation coefficient. Data in parentheses are 95% confidence intervals

Table S3. Interobserver agreement for measurements of CEST metrics in high grade gliomas

|  | ICC (95CI%) | P |
| --- | --- | --- |
| amide | 0.966(0.894-0.989) | <0.001 |
| NOE | 0.971(0.909-0.991) | <0.001 |
| amine | 0.963(0.885-0.988) | 0.001 |
| MT | 0.981(0.941-0.994) | 0.002 |
| DS | 0.976(0.926-0.992) | <0.001 |
| pH | 0.943(0.822-0.982) | 0.001 |
| MTR_3.5_ | 0.956(0.863-0.986) | 0.001 |

ICC interclass correlation coefficient. Data in parentheses are 95% confidence intervals

Table S4. Binary Logistic Regression Model for glioma grading andIDH genotyping

|  | Binary Logistic Regression Model | AUC |
| --- | --- | --- |
| Grade | Grade III=1/(1+exp(115.689*pH_weighted(mean)+8.952*DS(mean)- | 0.80 |
| II vs III | 9.403*MT(90th pc)-4.606) | (0.64-0.91) |
| Grade  III vs  IV | Grade IV=1/(1+exp(84.118*amide(75th pc)+64.192*NOE(75th  pc)+9.101*MT(75th pc)+48.029*ph_weighted(75th pc)-  16.398*DS(mean)+3.689) | 0.83  (0.74-0.90) |
| IDH- |  |  |
| wtvs | IDH-wt=1/(1+exp(83.390*amide(median)+26.036*NOE(10th | 0.84 |
| IDH- mt | pc)+80.987*ph_weighted(median)-19.689*MTR3.5(25th pc)-4.428) | (0.77-0.90) |

Data in parentheses are 95% confidence intervals.


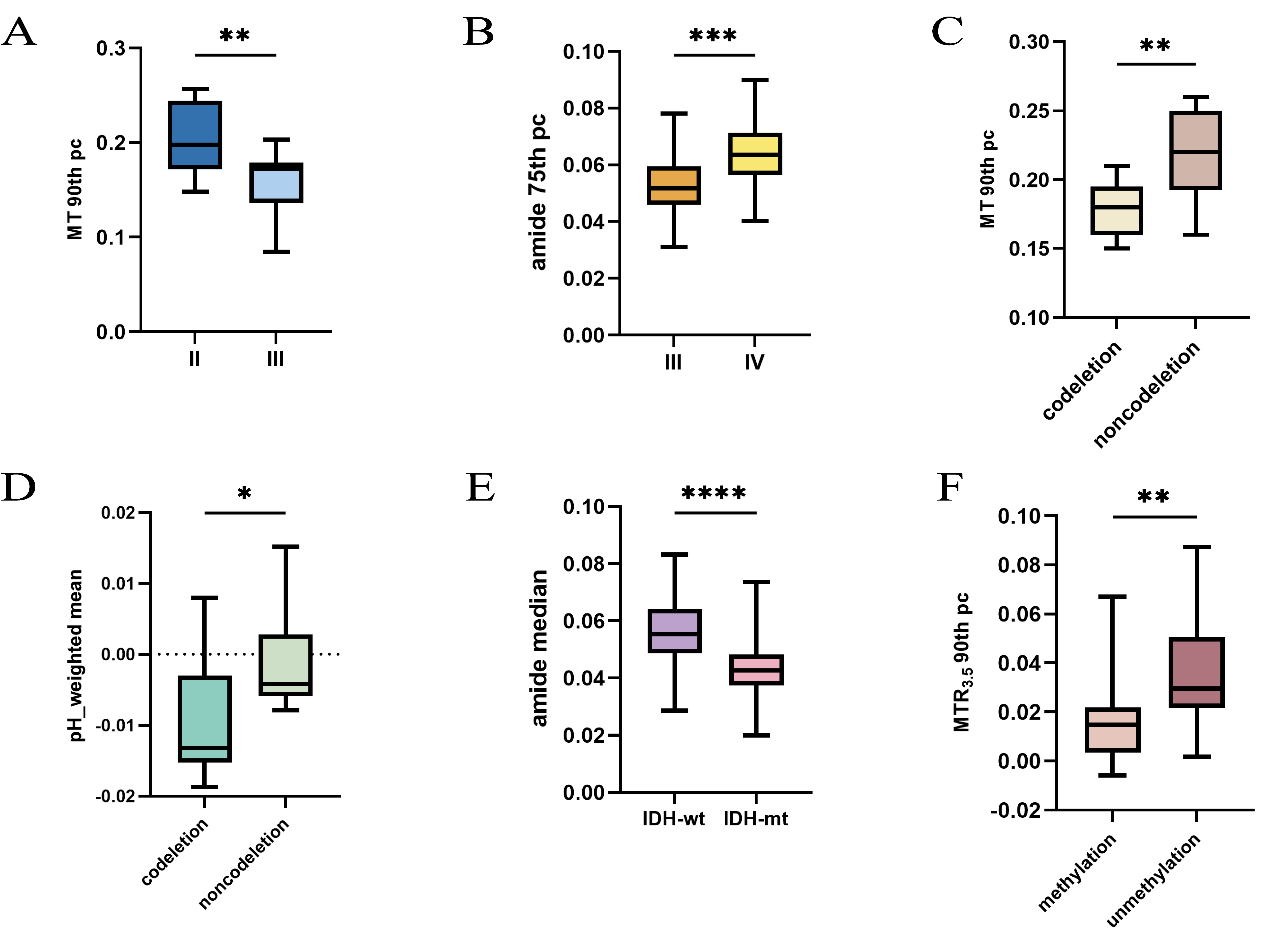


Figure S1. (A) CEST metric for differentiating between grade II and III. (B) CEST metric for differentiating between grade III and IV. (C) CEST metric for identifying between 1p/19q codeletion status within grade II gliomas. (D) CEST metric for identifying 1p/19q codeletion status within grade III gliomas. (E) CEST metric for identifying IDH mutation status. (F) CEST metric for identifying MGMT promoter methylation status.
